# Supplementary material for: Common transthyretin-derived amyloid fibril structures in patients with hereditary ATTR amyloidosis
Source: Nat Commun. 2023 Nov 22;14:7623. doi: 10.1038/s41467-023-43301-3 (PMC10665346; doi:10.1038/s41467-023-43301-3)
Supplement: Supplementary file 1 — Supplementary Information [file 41467_2023_43301_MOESM1_ESM.pdf]

Supplementary Information

Common transthyretin-derived amyloid fibril structures in patients with hereditary ATTR amyloidosis

Maximilian Steinebrei[1], Julian Baur[1], Anaviggha Pradhan[1], Niklas Kupfer[1], Sebastian Wiese[2], Ute Hegenbart[3], Stefan O. Schönland[3], Matthias Schmidt[1] & Marcus Fändrich[1]

Affiliations:

1. Institute of Protein Biochemistry, Ulm University, Helmholtzstrasse 8/1, Ulm D-89081, Germany
2. Core Unit Mass Spectrometry and Proteomics, Medical Faculty, Ulm University, Ulm D-89081, Germany
3. Medical Department V, Amyloidosis Center, Heidelberg, University Hospital Heidelberg, Im Neuenheimer Feld 400, Heidelberg D-69120, Germany

Supplementary Tables

Supplementary Table 1

**Mutational Variant**	**V20I**	**G47E**	**V122I**
**Acceleration voltage (kV)**	300	300	300
**Magnification**	x 105,000	x 130,000	x 130,000
**Defocus range (µm)**	-1.0 to -2.0	-0.9 to -1.8	-1.2 to -2.5
**Dose rate (e^-/Å/s)**	~15	~4	~3-4
**Exposure time (s)**	1.2	9	10
**Total electron dose (e^-/Å)**	40.33	~41.63	57.70
**Pixel size (Å)**	0.822	0.934	1.04
**Box size (pixel)**	256	256	256
**Inter box distance (Å)**	21.04	23.91	26.62
**Micrographs acquired**	3,954	12,067	2,068
**Number of extracted segments**	145,213	502,445	191,535
**Number of segments after 2D classification**	145,213	502,445	191,535
**Number of segments after 3D classification**	56,590	184,025	87,568
**Resolution, 0.143 FSC criterion (Å)**	3.39	2.37	2.99
**Map sharpening B-Factor (Å2)**	-69.83	-28.84	8.04
**Helical rise (Å)**	4.82	4.81	4.81
**Helical twist (°)**	-1.29	-1.24	-1.24

Supplementary Table 1.

Data collection and reconstruction parameters.

Supplementary Table 2

Mutational Variant	V20I	G47E	V122I
**Model resolution (Å)**			
**FSC threshold 0.143**	3.15	2.35	3.99
**FSC threshold 0.5**	4.59	2.60	3.34
**Model composition**			
**Non-hydrogen atoms**	2,157	4,308	4,314
**Protein residues**	276	522	522
**Ligands**	0	0	0
**B factors (Å2)**			
**Protein**	93.40	93.93	64.01
**Ligand**	-	-	-
**R.m.s. deviations**			
**Bond lengths (Å)**	0.013	0.013	0.013
**Bond angles (°)**	1.744	1.771	1.688
**Validation**			
**MolProbity score**	0.65	0.61	0.61
**Clashscore**	0.23	0.12	0.12
**Poor rotamers (%)**	0.00	0.00	0.00
**Ramachandran plot**			
**Favored (%)**	97.73	97.73	97.73
**Allowed (%)**	2.27	2.27	2.27
**Disallowed (%)**	0	0	0

Supplementary Table 2.

Modelling parameters.

Supplementary Table 3

Peak No.	Experimental mass (Da)	Protein Variant	Assigned fragment	Theoretical mass (Da)
1	3,600.9	WT	11-43	3,600.9
2	3,684.9	WT	89-122	3,684.8
3	3,818.0	WT	76-109	3,817.9
4	3,832.0	WT	9-43	3,832.0
5	3,848.0	WT	75-108	3,847.9
6	7,865.0	WT	58-127	7,864.9
7	8,091.1	WT	52-124	8,091.0
8	8,172.1	WT	55-127	8,172.1
		WT	54-126	8,172.1
9	8,283.2	V20I	17-91	8,283.1
		V20I	19-93	8,283.1
10	8,301.2	WT	54-127	8,301.2
11	8,358.2	WT	53-127	8,358.2
12	8,408.2	WT	7-82	8,408.2
		WT	49-124	8,408.1
13	8,429.2	WT	37-112	8,429.1
14	8,445.3	WT	51-126	8,445.2
		WT	52-127	8,445.2
15	8,517.3	WT	21-97	8,517.2
16	8,574.3	WT	51-127	8,574.3
17	8,645.3	V20I	12-89	8,645.4
18	8,661.3	WT	50-127	8,661.3
19	8,680.3	WT	4-83	8,680.4
		WT	46-124	8,680.3
20	8,718.3	WT	27-104	8,718.3
21	8,762.4	WT	7-86	8,762.4
		WT	49-127	8,762.3
22	8,776.4	V20I	7-86	8,776.5
23	8,890.5	WT	48-127	8,890.4

24	8,898.4	WT	44-124	8,898.4
25	8,947.5	WT	47-127	8,947.4
26	9,034.5	WT	46-127	9,034.5
27	9,105.5	WT	45-127	9,105.5
28	9,196.6	WT	40-122	9,196.5
		WT	41-123	9,196.5
29	9,252.6	WT	44-127	9,252.6

Supplementary Table 3

TTR fragments constituting the fibril proteins of patient V20I.

The peak number refers to the mass spectrometric peaks observed in figure 6. The peaks were assigned to WT or mutant TTR as indicated in the table.

Supplementary Table 4

Peak No.	Experimental mass (Da)	Protein Variant	Assigned fragment	Theoretical mass (Da)
1	3,503.9	WT	11-42	3,503.9
		WT	12-43	3,503.9
2	3,519.9	WT	4-37	3,519.9
3	3,600.9	WT	11-43	3,600.9
4	3,670.9	G47E	43-75	3,670.8
5	3,818.0	WT	76-109	3,817.9
6	4,330.2	WT	46-84	4,330.2
		WT	47-85	4,330.2
7	7,864.9	WT	58-127	7,864.9
		WT	27-96	7,864.9
8	8,357.2	G47E	18-92	8,357.1
9	8,428.2	WT	35-110	8,428.1
10	8,445.2	WT	51-126	8,445.2
		WT	52-127	8,445.2
11	8,501.2	G47E	37-112	8,501.1
12	8,661.3	WT	50-127	8,661.3
13	8,717.3	WT	32-109	8,717.3
14	8,762.4	WT	47-125	8,762.3
		WT	49-127	8,762.3
		WT	7-86	8,762.4
15	8,880.5	WT	4-85	8,880.5
16	9,034.5	WT	46-127	9,034.5
17	9,049.5	G47E	4-86	9,049.5
18	9,105.5	WT	45-127	9,105.5
19	9,162.5	WT	24-105	9,162.5
20	9,177.6	G47E	45-127	9,177.5
21	9,252.6	WT	44-127	9,252.6
22	9,308.6	G47E	28-110	9,308.6
23	9,324.6	G47E	44-127	9,324.6

Supplementary Table 4.

TTR fragments constituting the fibril proteins of patient G47E.

The peak number refers to the mass spectrometric peaks observed in figure 6. The peaks were assigned to WT or mutant TTR as indicated in the table.

Supplementary Table 5

Peak No.	Experimental mass (Da)	Protein Variant	Assigned fragment	Theoretical mass (Da)
1	3,503.9	WT	11-42	3,503.9
		WT	12-43	3,503.9
2	3,600.9	WT	11-43	3,600.9
3	3,670.9	V122I	90-123	3,670.9
4	3,818.0	WT	76-109	3,817.9
5	4,330.2	WT	46-84	4,330.2
		WT	47-85	4,330.2
6	7,865.0	WT	58-127	7,864.9
7	7,879.0	V122I	58-127	7,879.0
8	8,171.1	WT	52-124	8,091.0
9	8,297.2	WT	20-94	8,297.1
		WT	16-90	8,297.1
10	8,445.3	WT	51-126	8,445.2
		WT	52-127	8,445.2
11	8,459.3	V122I	52-127	8,459.2
		V122I	51-126	8,459.2
12	8,661.3	WT	50-127	8,661.3
13	8,675.4	V122I	50-127	8,675.3
14	8,693.4	WT	9-87	8,693.4
15	8,718.4	WT	27-104	8,718.3
16	8,731.4	WT	18-96	8,731.3
17	8,762.4	WT	7-86	8,762.4
		WT	49-127	8,762.3
18	8,776.4	V122I	49-127	8,776.3
19	8,880.5	WT	4-85	8,880.5
20	8,961.5	V122I	47-127	8,961.5
21	9,034.5	WT	46-127	9,034.5
22	9,048.5	V122I	46-127	9,048.5
		WT	1-84	9,048.6

23	9,105.5	WT	45-127	9,105.5
24	9,119.6	V122I	45-127	9,119.5
25	9,210.6	V122I	40-122	9,210.5
		V122I	41-123	9,210.5
26	9,252.6	WT	44-127	9,252.6
27	9,266.7	V122I	44-127	9,266.6
		WT	30-112	9,266.6

Supplementary Table 5.

TTR fragments constituting the fibril proteins of patient V122I.

The peak number refers to the mass spectrometric peaks observed in figure 6. The peaks were assigned to WT or mutant TTR as indicated in the table.

Supplementary Figures and Figure legends

Supplementary Figure 1

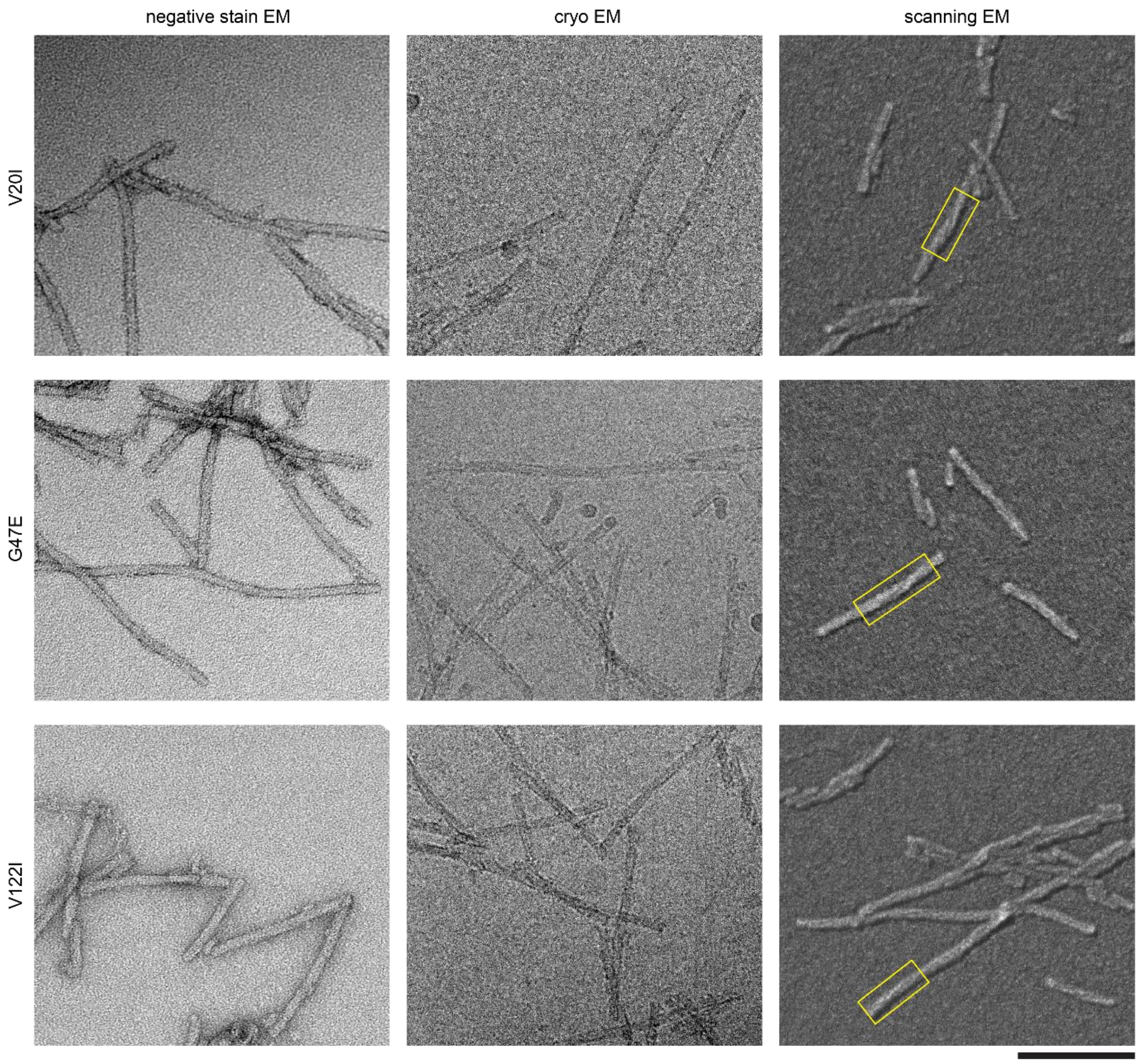

Supplementary Figure 1.

Electron micrographs of the extracted ATTR amyloid fibrils.

Upper row: images of the amyloid fibrils from patient V20. Central row: images of amyloid fibrils from patient G47E; bottom row: images of amyloid fibrils from patient V122I. Left column: negatively stained fibrils analyzed with TEM (representative for 100 recorded

micrographs). Central column: cryo-EM images of the ATTR fibrils (representative for ca 2,000 recorded micrographs). Right column: platinum side shadowed fibrils imaged with scanning electron microscopy (representative for 20 recorded micrographs). Boxes indicate fibrils with a discernible left-hand twist. All images are scaled to the same size. Scale bar: 100 nm.

Supplementary Figure 2

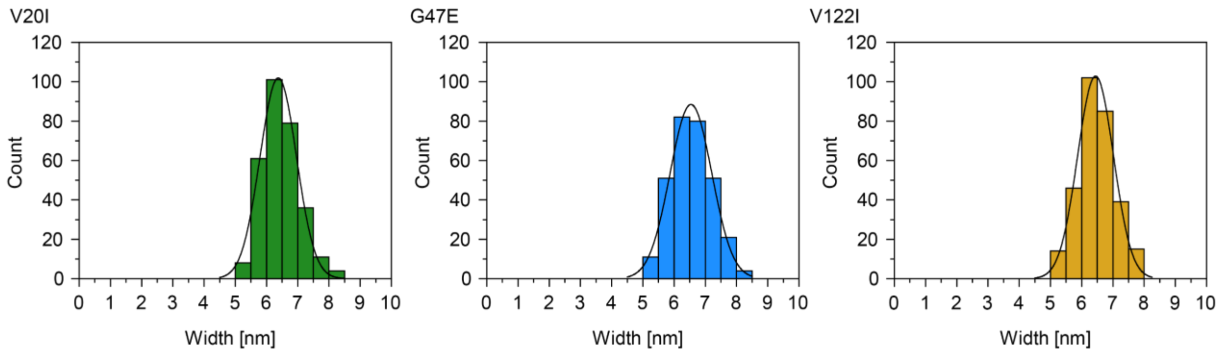

Supplementary Figure 2.

Width distribution of the three fibril samples.

Histograms of the fibril width measured from cryo-EM micrographs (n = 300 fibrils per patient) superimposed with a Gaussian function. The average width was determined at 6.5 ± 0.6 nm for the patient V20I fibrils, 6.6 ± 0.7 nm for the patient G47E fibrils and 6.5 ± 0.6 nm for the patient V122I fibrils.

Supplementary Figure 3

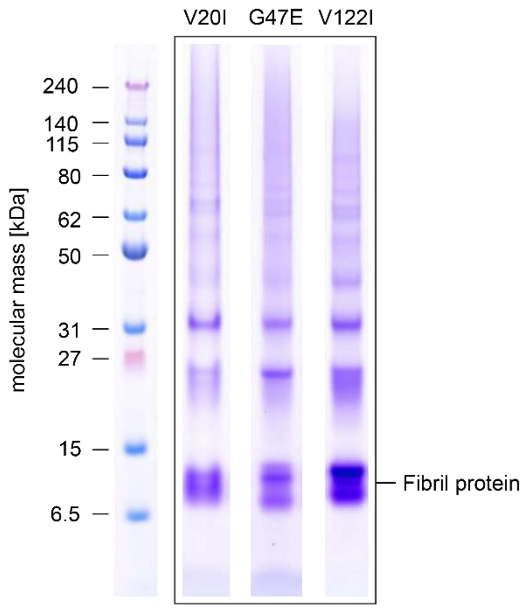

Supplementary Figure 3

Denaturing protein gel of the ATTR fibril proteins.

Coomassie-stained denaturing electrophoresis gel of the three amyloid fibril samples. The fibril proteins are found in a broad region between 6 kDa and 12 kDa.

Supplementary Figure 4

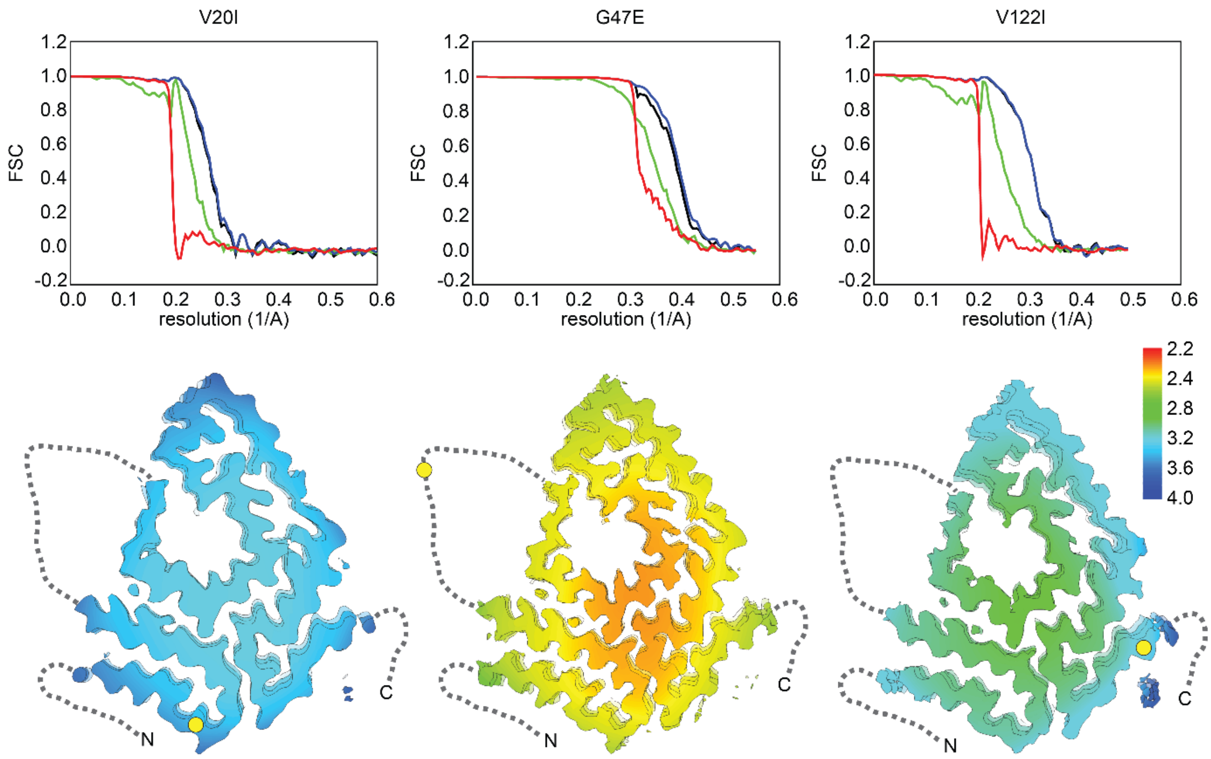

Supplementary Figure 4.

Resolution of the 3D maps.

Top row: FSCs between two half-maps for each 3D reconstruction. (black line = FSC corrected, green line = FSC unmasked maps, blue line FSC masked maps, red line = corrected FSC phase randomized masked maps). Bottom row: Cross sectional view of the 3D maps, showing a local resolution estimation in Å as provided by the color scale. The dotted line represents the unresolved part of the fibril protein, while the yellow dot indicates the location of mutation.

Supplementary Figure 5

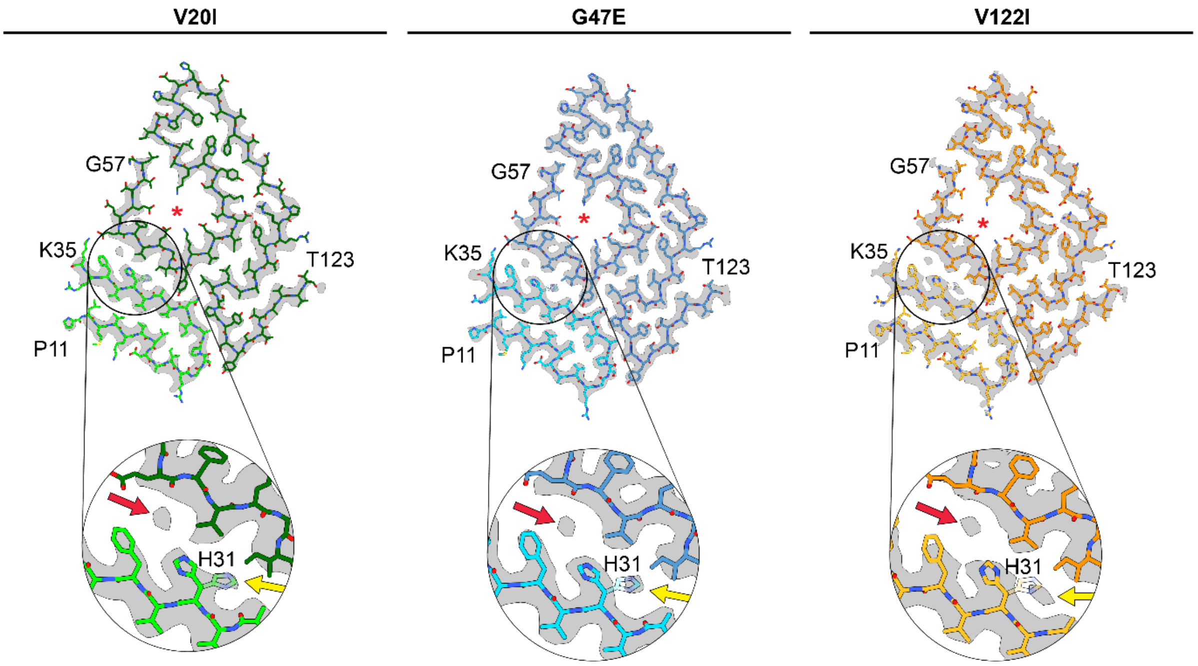

Supplementary Figure 5.

Extra density features in the 3D maps.

The yellow arrow points to the extra density close to position 31, which may arise from an alternative conformation for the adjacent histidine. The red arrow points to the density spot, which could not be assigned so far and may relate to a small molecular inclusion.

Supplementary Figure 6

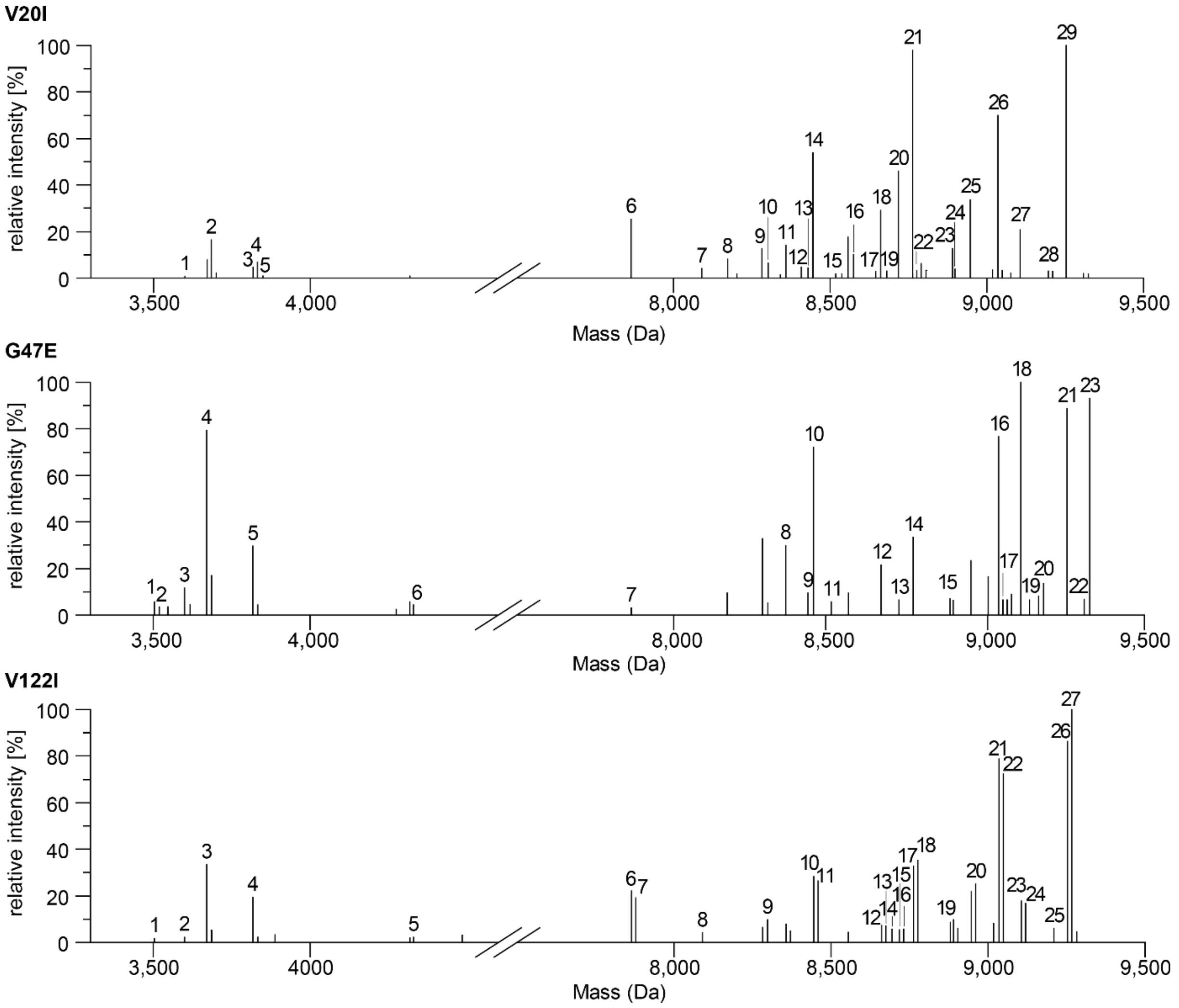

Supplementary Figure 6.

Mass spectra of extracted ATTR amyloid fibrils.

Deconvoluted mass spectra of the extracted fibril proteins from the three patients. The numbers above the peaks refer to the assigned TTR fragments (Supplementary Tables 3 to 5).

Supplementary Figure 7

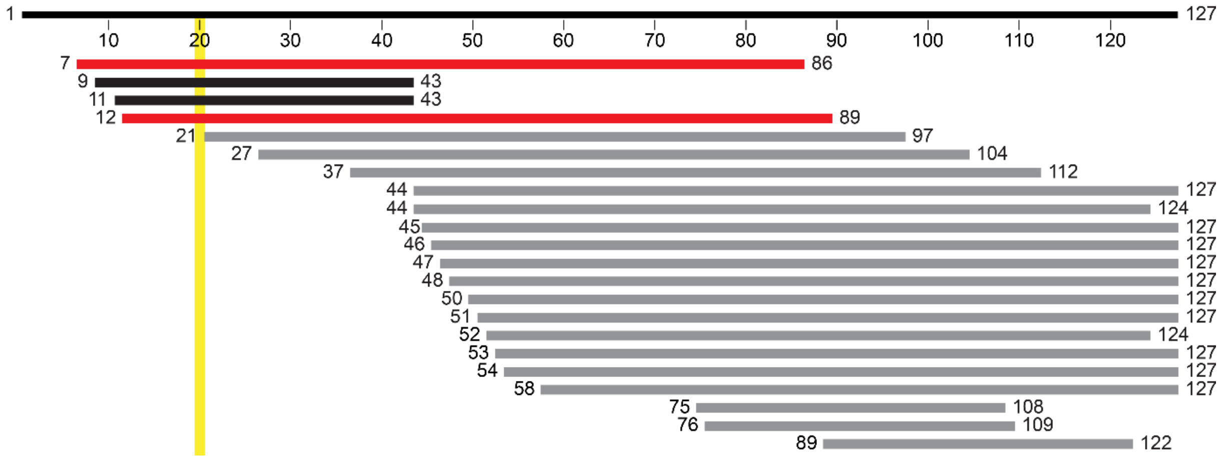

Supplementary Figure 7.

Schematic representation of the observed TTR fragments in the V20I sample.

Side-by-side view of the amino acid sequence of TTR V20I and the amyloid fibril protein fragments observed by mass spectrometry, which are shown as red, grey or black horizontal bars, below the sequence. The point mutation is shown by the yellow vertical bar. Red bars: fragments of TTR V20I. Black bars: fragments of WT TTR. Grey bars: fragments of either TTR V20I or WT TTR.

Supplementary Figure 8

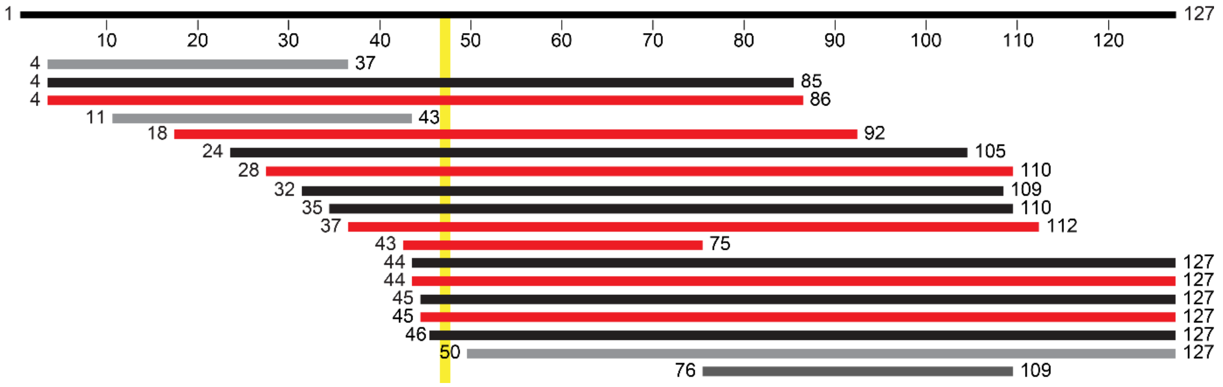

Supplementary Figure 8.

Schematic representation of the observed TTR fragments in the G47E sample.

Side-by-side view of the amino acid sequence of TTR G47E and the amyloid fibril protein fragments observed by mass spectrometry, which are shown as red, grey or black horizontal bars, below the sequence. The point mutation is shown by the yellow vertical bar. Red bars: fragments of TTR V20I. Black bars: fragments of WT TTR. Grey bars: fragments of either TTR V20I or WT TTR.

Supplementary Figure 9

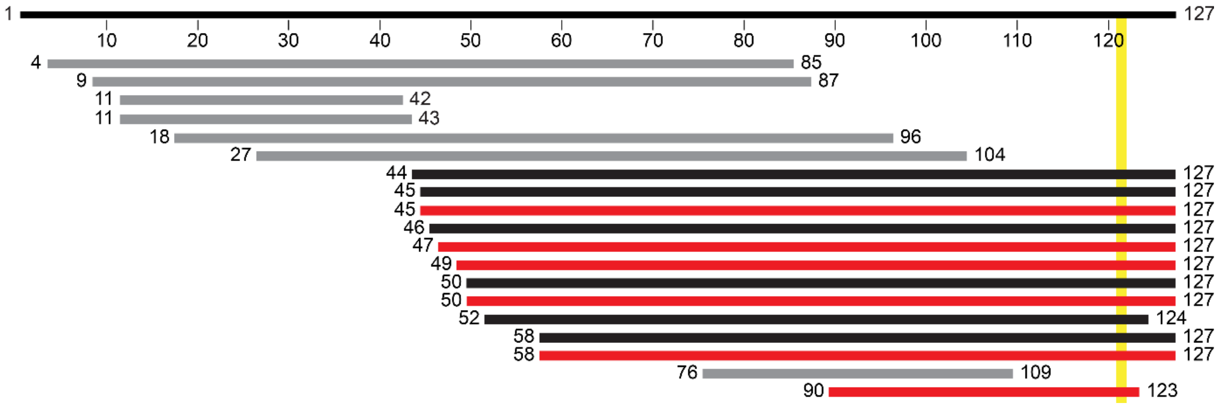

Supplementary Figure 9.

Schematic representation of the observed TTR fragments in the V122I sample.

Side-by-side view of the amino acid sequence of TTR V122I and the amyloid fibril protein fragments observed by mass spectrometry, which are shown as red, grey or black horizontal bars, below the sequence. The point mutation is shown by the yellow vertical bar. Red bars: fragments of TTR V20I. Black bars: fragments of WT TTR. Grey bars: fragments of either TTR V20I or WT TTR.

Supplementary Figure 10

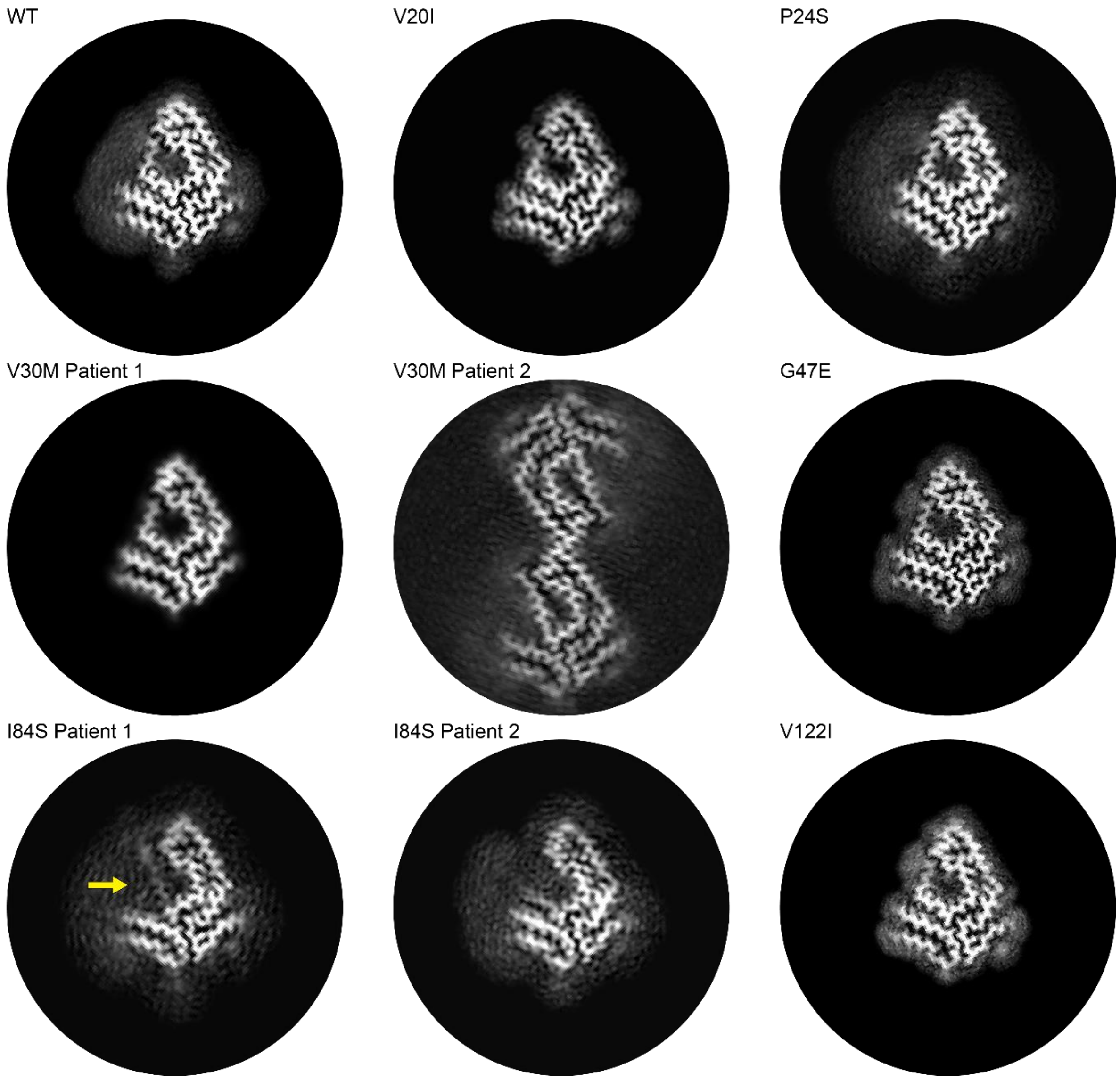

Supplementary Figure 10.

Comparison of available 3D maps of ATTR fibrils.

Each image shows a 5 Å thick fibril cross sectional slice of the reconstructed 3D map. Arrows point to residues G57-Y69, which show conformational heterogeneity. (WT EMD-15361, P24S EMD-26692, V30M patient 1 EMD-10150, V30M patient 2 EMD-12794, I84S patient 1 EMD-26685, I84S patient 2 EMD-27323)

Supplementary Figure 11

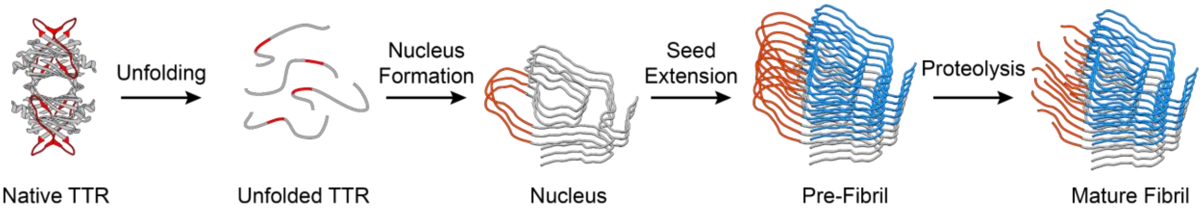

Supplementary Figure 11.

Schematic representation of the possible mechanism of ATTR fibril formation.

The reaction consists at least of the indicated steps and we assume that the nucleus formation step may be influenced by factors of the native cellular environment, such as lipids, glycosaminoglycans or proteases. Once the appropriate fibril seed is formed it may extend into a fibril, which becomes cleaved after the seed extension step.
