# Supplementary material for: Common transthyretin-derived amyloid fibril structures in patients with hereditary ATTR amyloidosis
Source: Nat Commun. 2023 Nov 22;14:7623. doi: 10.1038/s41467-023-43301-3 (PMC10665346; doi:10.1038/s41467-023-43301-3)
Supplement: Supplementary file 3 — Reporting Summary [file 41467_2023_43301_MOESM3_ESM.pdf]

nature portfolio

Corresponding author(s): Steinebrei

Last updated by author(s): 2023.10.19

Reporting Summary

Nature Portfolio wishes to improve the reproducibility of the work that we publish. This form provides structure for consistency and transparency in reporting. For further information on Nature Portfolio policies, see our Editorial Policies and the Editorial Policy Checklist.

Statistics

For all statistical analyses, confirm that the following items are present in the figure legend, table legend, main text, or Methods section.

n/a	Confirmed	
☐	☒	The exact sample size (*n*) for each experimental group/condition, given as a discrete number and unit of measurement
☐	☒	A statement on whether measurements were taken from distinct samples or whether the same sample was measured repeatedly
☒	☐	The statistical test(s) used AND whether they are one- or two-sided<br>*Only common tests should be described solely by name; describe more complex techniques in the Methods section.*
☒	☐	A description of all covariates tested
☒	☐	A description of any assumptions or corrections, such as tests of normality and adjustment for multiple comparisons
☒	☐	A full description of the statistical parameters including central tendency (e.g. means) or other basic estimates (e.g. regression coefficient) AND variation (e.g. standard deviation) or associated estimates of uncertainty (e.g. confidence intervals)
☒	☐	For null hypothesis testing, the test statistic (e.g. *F*, *t*, *r*) with confidence intervals, effect sizes, degrees of freedom and *P* value noted<br>*Give P values as exact values whenever suitable.*
☒	☐	For Bayesian analysis, information on the choice of priors and Markov chain Monte Carlo settings
☒	☐	For hierarchical and complex designs, identification of the appropriate level for tests and full reporting of outcomes
☒	☐	Estimates of effect sizes (e.g. Cohen's *d*, Pearson's *r*), indicating how they were calculated

Our web collection on statistics for biologists contains articles on many of the points above.

Software and code

Policy information about availability of computer code

Data collection	Serial EM v3.9.0beta
Data analysis	RELIONv3.1, CTFfind 4.1, IMOD v4.9.4, Coor v0.9.1, PHENIX v1.2, ImageJ v1.5.2p, UCSF Chimera v1.16 + v.1.17, XCalibur 2.2 SP1.48, mMass v5.50

For manuscripts utilizing custom algorithms or software that are central to the research but not yet described in published literature, software must be made available to editors and reviewers. We strongly encourage code deposition in a community repository (e.g. GitHub). See the Nature Portfolio guidelines for submitting code & software for further information.

Data

Policy information about availability of data

All manuscripts must include a data availability statement. This statement should provide the following information, where applicable:

- Accession codes, unique identifiers, or web links for publicly available datasets
- A description of any restrictions on data availability
- For clinical datasets or third party data, please ensure that the statement adheres to our policy

The reconstructed cryo-EM maps were deposited in the Electron Microscopy Data Bank with the accession code EMD-17736 (ATTRV20I), EMD-17737 (ATTRG37E) and EMD-17738 (ATTRV122I). The coordinates of the fitted atomic model were deposited in the Protein Data Bank under the accession code 8PKE (ATTRV20I), 8PKF (ATTRG37E) and 8PKG (ATTRV122I). The data that support the findings of this study are available from the corresponding authors upon reasonable request. The source data underlying Supplementary Fig. 1, Supplementary Fig. 2, Supplementary Fig. 3, Supplementary Fig. 4 and Supplementary Fig. 5 are provided as a Source

Data file.

Research involving human participants, their data, or biological material

Policy information about studies with human participants or human data. See also policy information about sex, gender (identity/presentation), and sexual orientation and race, ethnicity and racism.

Reporting on sex and gender	This study included tissue samples from one female and two male individuals.We could not identify sex and/or gender related differences in our findings
Reporting on race, ethnicity, or other socially relevant groupings	Not relevant
Population characteristics	Patients Sex: Male, Female, Male; Age: 56, 56, 51; TTR mutation: V20I, G47E, V122I
Recruitment	The individuals were selected based on clinical findings. All patients were diagnosed with hereditary ATTR amyloidosis and needed heart transplantation. The patients were treated within the heart transplant program of the University hospital Heidelberg.
Ethics oversight	The study was approved by the ethical committees of the University of Heidelberg (S-123/2006) and of Ulm University (103/21). Informed consent for publication of patient details was obtained from the patient, as well as consent for the use of these samples for scientific research.

Note that full information on the approval of the study protocol must also be provided in the manuscript.

Field-specific reporting

Please select the one below that is the best fit for your research. If you are not sure, read the appropriate sections before making your selection.

[x] Life sciences [ ] Behavioural & social sciences [ ] Ecological, evolutionary & environmental sciences

For a reference copy of the document with all sections, see nature.com/documents/nr-reporting-summary-flat.pdf

Life sciences study design

All studies must disclose on these points even when the disclosure is negative.

Sample size	The sample size is the determined number of particle images during cryo-EM imaging. The electron microscopy datasets included 3954 micrographs with 145 213 helical segments (V20I), 12067 micrographs with 502 445 helical segments, and 2,068 micrographs with 191 535 helical segments
Data exclusions	No data were excluded
Replication	Each reconstruction is based on a single patients specific dataset. In each case more then 10 different reconstructions lead to the same result, showing the reproducibility.
Randomization	The data shown represent single case studies for each genetic variant, therefore randomization is not relevant to study.
Blinding	The classification of the particle images were performed by computer and therefore objectively. Blinding was not relevant for the purification and sample characterization as there was only one type of fibril sample

Reporting for specific materials, systems and methods

We require information from authors about some types of materials, experimental systems and methods used in many studies. Here, indicate whether each material, system or method listed is relevant to your study. If you are not sure if a list item applies to your research, read the appropriate section before selecting a response.

Materials & experimental systems

n/a	Involved in the study
☒	☐ Antibodies
☒	☐ Eukaryotic cell lines
☒	☐ Palaeontology and archaeology
☒	☐ Animals and other organisms
☒	☐ Clinical data
☒	☐ Dual use research of concern
☒	☐ Plants

Methods

n/a	Involved in the study
☒	☐ ChIP-seq
☒	☐ Flow cytometry
☒	☐ MRI-based neuroimaging
